# Infection related catheter complications in patients undergoing prone positioning for acute respiratory distress syndrome: an exposed/unexposed study

**DOI:** 10.1186/s12879-021-06197-2

**Published:** 2021-06-07

**Authors:** Guillaume Louis, Thibaut Belveyre, Audrey Jacquot, Hélène Hochard, Nejla Aissa, Antoine Kimmoun, Christophe Goetz, Bruno Levy, Emmanuel Novy

**Affiliations:** 1 Intensive Care Unit, Metz-Thionville Regional Hospital, Mercy Hospital, 1 allée de Château, 57085 Metz, France; 2 Medical intensive Care Unit, University Hospital of Nancy, Brabois, France; 3 Department of Bacteriology, Metz-Thionville Regional Hospital, Mercy Hospital, Metz, France; 4 Department of Bacteriology, University Hospital of Nancy, Nancy, France; 5 Clinical Research Support Unit, Metz-Thionville Regional Hospital, Metz, France

**Keywords:** Colonization, Catheter-related infection, ARDS, Prone positioning

## Abstract

**Background:**

Prone positioning (PP) is a standard of care for patients with moderate–severe acute respiratory distress syndrome (ARDS). While adverse events associated with PP are well-documented in the literature, research examining the effect of PP on the risk of infectious complications of intravascular catheters is lacking.

**Method:**

All consecutive ARDS patients treated with PP were recruited retrospectively over a two-year period and formed the exposed group. Intensive care unit (ICU) patients during the same period without ARDS for whom PP was not conducted but who had an equivalent disease severity were matched 1:1 to the exposed group based on age, sex, centre, length of ICU stay and SAPS II (unexposed group). Infection-related catheter complications were defined by a composite criterion, including catheter tip colonization or intravascular catheter-related infection.

**Results:**

A total of 101 exposed patients were included in the study. Most had direct ARDS (pneumonia). The median [Q1–Q3] PP session number was 2 [1–4]. These patients were matched with 101 unexposed patients. The mortality rates of the exposed and unexposed groups were 31 and 30%, respectively. The incidence of the composite criterion was 14.2/1000 in the exposed group compared with 8.2/1000 days in the control group (*p* = 0.09). Multivariate analysis identified PP as a factor related to catheter colonization or infection (*p* = 0.04).

**Conclusion:**

Our data suggest that PP is associated with a higher risk of CVC infectious complications.

## Introduction

Prone positioning (PP) has become a standard of care for acute respiratory distress syndrome (ARDS) [1]. It is recommended that PP is begun in the first hours of moderate–severe ARDS, which is diagnosed by a PaO_2_/FiO_2_ ratio of ≤150 mmHg; once initiated, PP should be continued for at least 16 h [1, 2]. A 2018 APRONET study showed that the frequency of PP application for moderate–severe ARDS in intensive care units (ICUs) has steadily increased [3].

PP is known to be associated with multiple adverse events, including skin lesions (pressure sores), hemodynamic instability, airway obstruction, transient desaturation, displacement of endotracheal tubes, and incidental loss of venous access [4–10].

Critically ill patients with ARDS have high exposure to central venous catheters (CVCs) [5, 11]. We hypothesised that manipulations related to PP placement, limited access to the CVC dressing during PP, increased frequency of wound dressing, and extended duration of CVC exposure could promote infection-related catheter complications. To the best of our knowledge, the effect of PP on the risk of infection-related catheter complications has not been studied to date.

The incidence of infection-related CVC complications can be decreased by improving the quality of care [12–14]; therefore, determining the populations at risk of this complication is highly important [12]. Therefore, we tested whether PP is a risk factor for catheter tip colonization (CTC), catheter-related clinical sepsis (CRCS) and catheter-related bloodstream infections (CRBSI) by assessing the incidence of infection-related CVC complications in PP-treated ARDS patients (PP-exposed group) and patients without ARDS who did not undergo PP but who had similar disease severity.

## Material and methods

### Study design

This retrospective observational exposed/unexposed matched study was conducted in medical ICUs in two regional hospitals (Metz and Nancy) in France. All consecutive eligible patients who underwent PP for moderate–severe ARDS (exposed group) were matched 1:1 with control patients without ARDS who were not exposed to PP but who had similar disease severity at admission (as measured by the Simplified Acute Physiology Score [SAPS] II; unexposed group). Both ICUs were similar in terms of current practice regarding intravascular catheter-related infections prevention and catheter use [13, 15]. Maximal sterile barrier precautions and ultrasound guidance when placing CVCs were mandatory. Povidone-iodine in alcohol was used to prepare the skin prior to catheter insertion, and the use of transparent semi-permeable dressing (with no antiseptic) with no systematic change in dressing was recommended. Regarding the PP procedure, both centres followed guidelines for PP placement that conformed to the recommendations by Guerin et al. *[*1*]*. Moreover, in the two centres, all catheter tips were sent to a laboratory of bacteriology for conventional culture after removal for all patients in routine practice.

### Study population recruitment

Adult (≥18 years) patients were enrolled in the study between 1 January 2014 and 31 December 2015. This was a stable period of practice in terms of the PP procedure and the use of antiseptics, dressings, and catheter materials. Patients admitted into the ICU for moderate–severe ARDS and treated with PP during ARDS according to Berlin 2012 criteria were included in the exposed group [16]. Patients were excluded if they had previously undergone PP in another ICU before admission into one of the two participating centres, if they lacked a venous catheter or if bacteriological culture results were unavailable after catheter removal. Patients were considered for inclusion in the unexposed group if they were admitted to the ICUs during the same period (2014–2015) and were not treated with PP during their ICU stay. Likewise, controlled patients who lacked venous catheters or bacteriological culture results after catheter removal were excluded. Eligible unexposed patients were included in the unexposed group if they each matched an exposed patient in terms of age, sex, year of hospitalisation, centre, disease severity (SAPS II) at admission and length of ICU stay. For this purpose, we used clusters of ages (18–40, 41–59, 60–79 and > 79 years) and length of ICU stay (< 7, 7–13, 14–20 and > 20 days). With regard to severity, the SAPS II score was used without age to avoid excessive matching, as age was already a matching factor. Only short-term venous catheters were considered (CVC or dialysis catheter).

### Primary and secondary outcome variables

According to international definitions [17], catheter colonization was a positive quantitative catheter-tip culture that yields ≥ 10^3^ CFU/mL, according to the method of Brun-Buisson. Catheter-related bloodstream infection was *either* as one positive blood culture obtained from peripheral vein and clinical manifestation of infection and CTC *or* positive central and peripheral blood cultures with the same microorganism, with a central/peripheral positive blood culture time-lag > 2 h, with central blood cultures being positive earlier than the peripheral ones. Catheter-related clinical sepsis was clinical manifestation of infection that disappears within 48 h of catheter removal and a positive catheter tip culture and no other obvious treated source of infection. Catheter-related infection was defined as the combination of CRBSI and CRCS.

Due to the low rates of catheter-related infectious in the two centres, a large number of patients would have been needed to reach statistical significance. For this reason, the primary outcome variable was incidence of a composite criterion, including CTC or intravascular catheter-related infection. The results were expressed as the rate of incidence for 1000 catheter days.

The secondary outcomes were risk factors related to catheter colonization or infection. The types of microorganisms isolated were also noted. We intentionally excluded Coagulase-negative *Staphylococcus* species from the analysis given the limited pathogenicity of this pathogen.

### Data collection

Patients were screened for moderate–severe ARDS using the French medico-administrative database (PMSI). Patients were candidates for inclusion if their stay contained a diagnostic code for ARDS (J80 in the International Classification of Diseases, 10th revision) and the following medical acts (*Classification commune des actes médicaux*, 48th revision): GLLD004 (mechanical ventilation PEEP ≥6 cmH_2_0, FiO_2_ ≥ 60% and PP) and either EPLF002 (central venous access) or EPLF005 (dialysis catheter). The patients’ baseline characteristics and infection-related variables were collected from electronic health records, including microbiology and pharmacy records. The baseline characteristics that were collected included demographic data and SOFA score, SAPS II score, organ failures and comorbidities at admission. Patients were considered immunocompromised if they had diabetes, neoplasia or neutropenia/aplasia or were receiving immunosuppressive therapy. The following data on potential risk factors for catheter-related infection were also collected: number of catheters per patient, duration of catheterisation, vascular access site, whether the patient received antibiotic treatment at the time of catheter insertion and parenteral nutrition.

### Ethics

The study was approved by the Ethics Committee of the French Intensive Care Society (record number CE SRLF 17–51) and was conducted according to the MR-003 reference methodology (record number 2061208) of the French National Commission on Information Technology and Liberties (CNIL). The Ethics Committee of the French Intensive Care Society waived the requirement of written informed consent. According to French laws on biomedical research, patients were notified about the anonym use of their healthcare data via an information letter and no written consent form was required for a retrospective study. None of the patients expressed any opposition to the use of their data. All methods were performed in accordance with the relevant guidelines and regulations.

This manuscript was written in accordance with the STROBE statement (www.strobe-statement.org) for the reporting of observational studies in epidemiology. The study was registered with ClinicalTrials.gov under identification number NCT 03405038.

### Sample size and statistical analysis

Based on a local French survey of nosocomial infections in adult ICU patients (https://www.santepubliquefrance.fr/maladies-et-traumatismes/infections-associees-aux-soins-et-resistance-aux-antibiotiques/infections-associees-aux-soins/documents/rapport-synthese/surveillance-des-infections-nosocomiales-en-reanimation-adulte.-reseau-rea-raisin-france-resultats-2015) and local CVC monitoring of our ICUs, we hypothesised that the exposed and unexposed groups would have colonization rates of 8 and 4 per 1000 catheter days, respectively. The sample size calculation was based on the colonization rate with the postulate that there is a good correlation between colonization and catheter infection [18]. To statistically confirm this difference, 1000 ICU catheter days were included in each group. Given that ICU patients’ mean length of stay is 10 days [19], the required sample size was defined as at least 100 patients per group.

The prone and supine groups were compared in terms of qualitative and quantitative variables using Mac Nemar and signed-rank tests, respectively. Patients with or without catheter colonization or infection (primary outcome) were compared with a bivariate, then multivariate logistic regression. The significance level was set at 0.05. All analyses were conducted using SAS 9.3 (SAS Inst., Cary, NC).

## Results

### Study population

**Fig. 1:**
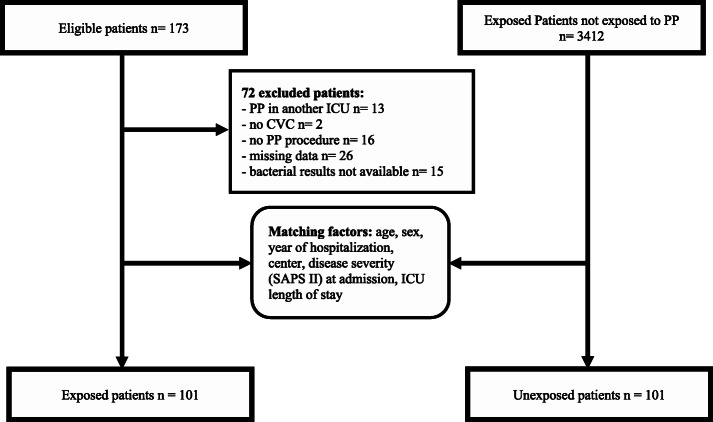
Flow chart. Abbreviations: PP: Prone positioning; ICU: Intensive Care Unit; SAPS II: Simplified Acute Physiology Score II; CVC: Central venous catheter

Between 1 January 2014 and 31 December 2015, 173 patients were treated for ARDS in two ICUs. Of these patients, 101 met the eligibility criteria. A flowchart of patients is presented in Fig. 1. The 101 patients with ARDS included in the study were then matched to 101 unexposed patients recruited from total admissions to the ICUs.

The main demographic and clinical characteristics of the cohort as a whole and the exposed and unexposed groups are presented in Table 1. The patients were mostly admitted for medical disorders (82%). The median (Q1–Q3) SAPS II and SOFA scores at admission were 54 (43–66) and 9 (8–12), respectively. Compared with the unexposed group, the exposed group had a significantly higher body mass index (30 [26–35] vs 27 (24–31), *p* = 0.01), a lower rate of renal replacement therapy (22% vs 38%, *p* = 0.02), a longer duration of mechanical ventilation (20 (12–29) vs 10 (3–19), *p* < 0.001) and a longer median length of ICU stay (23 (13–31) vs 16 (10–24) days, *p* = 0.004). The two groups did not differ significantly regarding the frequency of immunosuppressive conditions, patient origin before ICU admission, shock or duration of catecholamine infusion. The overall mortality of the cohort was 31%. The two groups did not differ significantly in terms of mortality (*p* = 0.76). The exposed and unexposed groups did not differ in the median number of catheters per patient (2 vs 2, *p* = 0.73), catheter insertion site (*p* = 0.81), or catheter utilisation (*p* = 0.12). Jugular access was the most commonly used route in both groups. The exposed patients were more likely to have a longer duration of catheterisation (19 vs 14) days (*p* = 0.049).

**Table 1:** Demographic and clinical characteristics at admission and clinical outcomes of the intensive care unit stay

Characteristics	Total cohort (***N*** = 202)	Exposed (prone) group (***N*** = 101)	Unexposed (supine) group (***N*** = 101)	Pair differences or discordances	p*
**Centre 1 (VS 2)**	90 (45)	45 (45)	45 (45)	0 / 0	1
**Gender (M)**	147 (73)	74 (73)	74 (73)	0 / 0	1
**Age (y)**	61 (48–68)	61 (46–68)	61 (52–70)	1 (−4–8)	0.63
**BMI (kg/m**^**2**^**)**	28 (25–34)	30 (26–35)	27 (24–31)	-1 (−8.5–3)	**0.003**
**SAPS II score**	54 (43–66)	54 (43–66)	53 (44–66)	0 (−5–6)	0.94
**SOFA score**	9 (8–12)	10 (8–12)	9 (8–11)	0 (−3–2)	0.43
**Immunosuppression**^a^	98 (49)	51 (51)	47 (47)	26 / 22	0.67
**Surgical admission (vs. medical)**	36 (18)	14 (14)	22 (22)	8 / 16	0.20
**Nosocomial patient origin (vs. community)**	98 (49)	49 (49)	49 (49)	25 / 25	1
**Catheterization duration (days)**	17 (8–26)	19 (9–27)	14 (8–25)	-1 (−13–6)	**0.049**
**Number of catheter per patient**	2 (1–3)	2 (1–2)	2 (1–3)	0 (−1–1)	0.73
**Catheter insertion site**					0.81
***Jugular***	148 (73)	76 (75)	72 (71)	21 / 25	
***Subclavian***	18 (9)	8 (8)	10 (10)	10 / 8	
***Femoral***	36 (18)	17 (17)	19 (19)	19 / 17	
**Catheter utilization**					0.12
***Dialysis***	37 (18)	24 (24)	13 (13)	12 / 23	
***Parenteral nutrition***	27 (13)	14 (14)	13 (13)	8 / 9	
***Other***	138 (68)	63 (62)	75 (74)	27 / 15	
**Mechanical ventilation duration (days)**	16 (7–25)	20 (12–29)	10 (3–19)	− 6 (− 15–-1)	**< 0.001**
**Catecholamine infusion duration (days)**	3 (0–6)	3 (0–6)	3 (0–6)	0 (−3–3)	0.88
**Shock**	132 (65)	66 (65)	66 (65)	20 / 20	1
**Renal replacement therapy**	60 (30)	22 (22)	38 (38)	11 / 27	**0.02**
**ICU length of stay**	18 (11–27)	23 (13–31)	16 (10–24)	−2 (−11–1)	**0.004**
**Mortality**	63 (31)	31 (30)	33 (33)	16 / 13	0.76

The data are expressed as number (%) or median (Q1–Q3). Discordant pairs are presented as positive in prone group and negative in supine group / negative in prone group and positive in supine group

*Abbreviations*: *BMI* Body Mass Index, *ICU* Intensive Care Unit, *SAPS II* Simplified Acute Physiology Score II, *SOFA* Sequential Organ Failure Assessment Score

*p values were obtained by comparing the exposed and unexposed groups by MacNemar or signed rank tests

^a^Immunocompromised conditions: diabetes, neoplasia, transplant, neutropenia/aplasia, immunosuppressive therapy

The main cause of ARDS in the exposed group was pneumonia (90%). ARDS was severe (PaO2/FIO2 ≤ 100 mmHg) in 70% of the cases. The median number of PP sessions per patient was 2 (2–4). The overall median duration of mechanical ventilation was 16 (7–25) days.

### Primary outcome

**Fig. 2:**
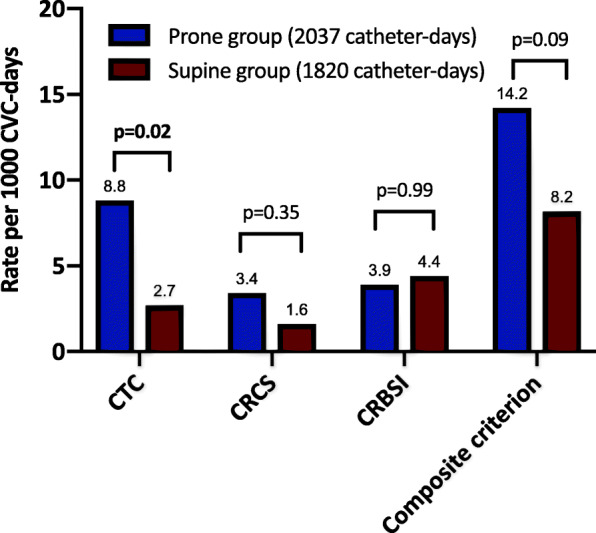
Incidences in the exposed and unexposed groups of central venous catheter (CVC) tip colonization (CTC), catheter-related clinical sepsis (CRCS), catheter-related bloodstream infection (CRBSI), and a composite outcome composed of CTC and/or CRCS and/or CRBSI

The total duration of catheterisation in the exposed and unexposed groups was 2037 and 1820 days, respectively. The incidences of the composite criterion were 14.2/1000 and 8.2/1000 CVC days, respectively (*p* = 0.09). The exposed group had a greater incidence of colonization (8.8/1000 and 2,7/1000 CVC days; *p* = 0.02) and a two-fold higher incidence of CRCS compared with the unexposed group (3.4/1000 vs 1.6/1000 CVC days), though the difference did not achieve statistical significance (*p* = 0.35). The two groups did not differ in terms of CRBSI (3.9/1000 vs 4.4/1000 CVC days, *p* = 0.99) (Fig. 2).

### Risk factors for CVC colonization or infection

Multivariate logistic regression of patients with one or more colonised or infected CVCs vs patients without colonised or infected CVCs showed that CVC colonization or infection was associated with the use of PP (OR 2.73, 95% CI [1.04–7.17], *p* = 0.04). The number of catheters per patient was associated with a decreased risk of catheter colonization or infection (OR 0.54, 95% CI [0.27–0.98], *p* = 0.03) though the duration of catheterisation was not associated with increased risk (*p* = 0.32). The variables “number of catheters” and “duration of catheterization” were significantly correlated (r = 0.6, *p* < 0.001). Therefore, the variable “number of catheters” was excluded from the multivariate model. The duration of catheterization was associated with catheter colonization or infection (*p* = 0.01) while all other significant results remained unchanged. The use of jugular or femoral access was associated with a higher risk of CTC or infection compared with subclavian access (OR 9.86, 95% CI [2.31–28.44], *p* = 0.005 and OR 6.43, 95% CI [1.53–19.51], *p* = 0.02, respectively) (Table 2).

**Table 2:** Factors related to a catheter colonization or infection

Factors	Catheter colonized or infected (***N*** = 44)	No colonization or infection (***N*** = 158)	Bivariate* p	Multivariate** OR (95% CI)	Multivariate** p
**Prone position (vs. supine)**	29 (66)	72 (46)	0.02	2.73 (1.04–7.17)	**0.04**
**Centre 1 (vs. 2)**	23 (52)	67 (42)	0.32	2.41 (0.93–6.30)	0.09
**Gender (Male)**	34 (77)	114 (72)	0.50	1.28 (0.44–3.72)	0.73
**Age (y)**	63 (53–73)	61 (47–68)	0.15	1.01 (0.97–1.04)	0.55
**BMI (kg/m**^**2**^**)**	28 (27–36)	27 (24–33)	0.14	1.06 (1.00–1.12)	0.09
**SAPS II score**	56 (45–69)	53 (43–66)	0.47	1.01 (0.99–1.04)	0.48
**Immunosuppression**	22 (50)	76 (48)	0.80	0.60 (0.24–1.52)	0.37
**Surgical admission (vs. medical)**	15 (34)	21 (13)	0.08	2.19 (0.70–6.93)	0.08
**Nosocomial patient origin (vs. community)**	27 (61)	71 (45)	0.08	2.49 (0.97–6.70)	0.09
**Catheterisation duration (days)**	16 (7–26)	17 (8–27)	0.75	0.99 (0.94–1.03)	0.32
**Number of catheter per patient**	1 (1–3)	2 (1–3)	0.25	0.54 (0.27–0.98)	**0.03**
**Catheter insertion site**			0.04		
**Jugular**	33 (75)	115 (73)		9.86 (2.31–28.44)	**0.005**
**Subclavian**	0 (0)	18 (11)		Ref.	**–**
**Femoral**	11 (25)	25 (16)		6.43 (1.53–19.51)	**0.02**
**Catheter utilisation**			0.57		
**Dialysis**	9 (20)	28 (18)		0.32 (0.06–1.27)	0.07
**Parenteral nutrition**	6 (14)	21 (13)		0.82 (0.18–3.71)	0.59
**Other**	29 (66)	109 (69)		Ref.	–
**Mechanical ventilation duration (days)**	22 (13–32)	15 (6–23)	0.03	1.05 (1.01–1.09)	**0.002**
**Cathecholamine infusion duration (days)**	4 (1–8)	3 (0–6)	0.29	0.95 (0.85–1.07)	0.34
**Shock**	31 (70)	101 (64)	0.30	1.43 (0.43–4.74)	0.53
**Renal replacement therapy**	23 (52)	37 (23)	0.03	14.93 (3.84–58.09)	**< 0.001**

The data were expressed as number (%) or median [Q1-Q3]

*Abbreviations*: *CVC* central venous catheter, *BMI* Body Mass Index, *OR* Odds Ratio, *CI* Confidence Intervals, *SAPS II* Simplified Acute Physiology Score II

*The exposed and unexposed groups were compared by using bivariate conditional logistic regression

**The multivariate conditional logistic regression involved comparing the patients with one or more colonized or infected catheters to patients without any colonized or infected catheters. It included matching variables (sex, age, center, and SAPS II score) and variables that achieved a *p* value of < 0.1 on the bivariate analyses in Tables 1 and 2

### Results of catheter cultures

During the study period, 440 CVCs (221 in the exposed group and 219 in the unexposed group) were cultured in both ICUs; of these, 95 (22%) were positive. The positive culture rate was 27% (61/221) in the exposed group vs 16% (34/219) in the unexposed group (*p* = 0.003).

The microorganisms recovered from CVC are listed in Table 3. The two groups did not differ significantly in terms of the frequencies of different microorganisms (*p* = 0.86). Coagulase-negative *Staphylococcus* species were more common in colonised catheters (51%), while *Enterobacteriaceae* species were more frequently isolated from CRCS (36%).

**Table 3:** Microorganisms recovered from the colonized and infected CVC

Microorganism	Exposed group (***N*** = 74)	Unexposed group (***N*** = 42)	***p*** value*
**Staphylococcus species**	30 (41)	18 (43)	0.86
*Staphylococcus aureus*	1 (1)	2 (5)	
*Staphylococcus epidermidis*	28 (38)	15 (36)	
*Staphylococcus haemolyticus*	1 (1)	1 (2)	
**Other GPB**	4 (5)	3 (7)	
*Enterococcus faecalis*	4 (5)	2 (5)	
*Enterococcus faecium*	0 (0)	1 (2)	
**Enterobacteriaceae**	21 (28)	13 (31)	
**Non fermenter GNB**	8 (11)	2 (5)	
**Other GNB**	1 (1)	0 (0)	
**GPB**	1 (1)	0 (0)	
**Polymorph bacterial flora**	3 (4)	3 (7)	
**Fungi**	5 (7)	1 (2)	

The data were expressed as number (%)

*Abbreviations*: *GPB* Gram-Positive Bacilli, *GNB* Gram-Negative Bacilli, *GPB* Gram-Positive Bacilli

*The exposed and unexposed groups were compared by using Fisher’s exact test

## Discussion

In this study, PP was associated with a higher risk of CVC colonization or infection (composite criterion). Moreover, the PP-exposed group had a significantly higher incidence of CVC tip colonization. This is the first study to assess CVC infections in patients with PP during ARDS.

The exposed patients were mainly patients with direct ARDS, the majority of whom with pneumonia. The mortality in this group was 30%, and the median length of mechanical ventilation was 20 days. This is consistent with other studies conducted in patients with moderate–severe ARDS [1, 8, 20, 21].

We compared our PP-exposed patients to control patients without ARDS who did not undergo PP. It was not possible to obtain a control population of ARDS patients who did not require PP, as all moderate–severe ARDS cases in both centres underwent PP. We also decided not to compare our exposed patients to patients with mild ARDS who did not require PP because the different degree of illness between the two groups could introduce bias. Instead, we identified a control group without ARDS with similar mortality and severity using SAPS II scores and clusters of age and length of ICU stay.

In both groups, the jugular was the most common CVC insertion site: 59% of the whole cohort had jugular access. In the present study, the subclavian insertion site was associated with less catheter colonization and infection compared with the jugular femoral insertion site. Strong data suggests that subclavian insertion is associated with fewer infectious complications than jugular or femoral insertion. Nevertheless, this site is also associated with more mechanical complications (pneumothorax) when the puncture is achieved without the use of ultrasound guidance [22]. Both centres in the present study systematically employed ultrasound-guided catheter insertion for the jugular site, which is why most patients in the present study had jugular access. This precluded the ability to draw conclusions in the case of ultrasound-guided subclavian puncture.

In both groups, coagulase-negative *Staphylococcus* species and *Enterobacteriaceae* were most commonly isolated. This is consistent with previous research [23].

We found that catheter replacement was associated with fewer infectious complications. To date, there is no evidence to support a replacement strategy on any schedule as an effective means of preventing catheter infection [24]. Nevertheless, the specific population of ARDS patients has not been investigated in previous studies.

We hypothesised that the PP-exposed patients had a higher incidence of CVC colonization or infection than the unexposed group because PP may complicate endobuccal aspiration or mouth care or hamper close monitoring of the CVC dressing. As CVCs are placed in relatively close proximity to the oral sphere and salivary secretions, these PP-related practical difficulties may elevate the risk of extraluminal contamination especially for the jugular insertion site. Recent studies and guidelines suggest that lengthening the time between CVC dressings is not associated with a higher incidence of catheter-related infection [25].

Several study limitations should be acknowledged. First, some ARDS cases may have been missed due to the retrospective nature of the study. However, this is unlikely to be a significant problem because we employed rigorous definitions of ARDS, catheter-related infection and colonization and examined both the medical records and microbiology database to identify the cases. We first sought to evaluate a population at risk of catheter-related infection, as additional measures related to this pathology (such as impregnated catheters) should be limited to selected populations. Thus, it is important to acknowledge the specificity of the two centres included in the present study, which resulted in a homogeneous population of direct and medical ARDS. In addition, practices associated with catheter-related infection prevention were similar, although the study period was anterior to the CLEAN trial [26]. Hence, the CHLORAPREP® device was not among the antiseptics used. This precludes the ability to draw conclusions regarding this method of catheter-related infection prevention, which is currently strongly recommended [17].

Our data could be considered relatively old. However, we believe that they are important in the current health context. In light of the coronavirus disease 2019 (COVID− 19) pandemic, PP is of crucial importance in treating severe ARDS patients [27].

Lastly, we intentionally chose a composite criterion to consider the diversity of manifestations of this specific pathological process in numerous clinical situations. We acknowledge that our composite criterion could constitute a limitation. We sought to determine the specific bounds related to PP to prevent catheter-related infection. This intermediate outcome was also chosen due to the low rate of catheter-related infection (colonization excluded) in both ICUs. Thus, the higher incidence of CVC colonization in the exposed patients may not translate into a higher incidence of catheter-related infection. Nevertheless, the exposed patients had a two-fold higher incidence of CRCS, although this difference did not achieve statistical significance, which could be due to the lack of CRCS in the two centres. Thus, although the results of the present study are interesting, further studies are needed to determine what measures may prevent CVC infections in PP patients (e.g., antiseptic devices or antimicrobial-coated catheters).

## Conclusions

The present study found that PP was associated with a higher risk of CVC infectious complications. This finding requires further investigation to yield broader conclusions. If the finding is confirmed by further studies, patients undergoing PP may benefit from additional measures to prevent CVC infections.

## Data Availability

The datasets used and/or analyzed during the current study are available from the corresponding author on reasonable request.

## Abbreviations

ARDS: Acute respiratory distress syndrome
CRBSI: Catheter-related bloodstream infection
CVC: Central venous catheter
CRCS: Catheter-related clinical sepsis
CTC: Catheter tip colonization
ICU: Intensive care unit
PP: Prone positioning
SOFA: Sequential-Related Organ Failure Assessment
SAPS II: Simplified Acute Physiology Score II

## References

[1] Guérin C, Reignier J, Richard J-C, Beuret P, Gacouin A, Boulain T, Mercier E, Badet M, Mercat A, Baudin O, Clavel M, Chatellier D, Jaber S, Rosselli S, Mancebo J, Sirodot M, Hilbert G, Bengler C, Richecoeur J, Gainnier M, Bayle F, Bourdin G, Leray V, Girard R, Baboi L, Ayzac L (2013). Prone positioning in severe acute respiratory distress syndrome. N Engl J Med.

[2] Fan E, Del Sorbo L, Goligher EC, Hodgson CL, Munshi L, Walkey AJ (2017). An official American Thoracic Society/European Society of Intensive Care Medicine/Society of Critical Care Medicine clinical practice guideline: mechanical ventilation in adult patients with acute respiratory distress syndrome. Am J Respir Crit Care Med.

[3] Guérin C, Beuret P, Constantin JM, Bellani G, Garcia-Olivares P, Roca O (2018). A prospective international observational prevalence study on prone positioning of ARDS patients: the APRONET (ARDS prone position network) study. Intensive Care Med.

[4] Park SY, Kim HJ, Yoo KH, Park YB, Kim SW, Lee SJ, Kim EK, Kim JH, Kim YH, Moon JY, Min KH, Park SS, Lee J, Lee CH, Park J, Byun MK, Lee SW, Rlee C, Jung JY, Sim YS (2015). The efficacy and safety of prone positioning in adults patients with acute respiratory distress syndrome: a meta-analysis of randomized controlled trials. J Thorac Dis.

[5] Sud S, Friedrich JO, Adhikari NKJ, Taccone P, Mancebo J, Polli F, Latini R, Pesenti A, Curley MAQ, Fernandez R, Chan MC, Beuret P, Voggenreiter G, Sud M, Tognoni G, Gattinoni L, Guérin C (2014). Effect of prone positioning during mechanical ventilation on mortality among patients with acute respiratory distress syndrome: a systematic review and meta-analysis. CMAJ..

[6] Lee JM, Bae W, Lee YJ, Cho Y-J (2014). The efficacy and safety of prone positional ventilation in acute respiratory distress syndrome: updated study-level meta-analysis of 11 randomized controlled trials. Crit Care Med.

[7] Taccone P, Pesenti A, Latini R, Polli F, Vagginelli F, Mietto C, Caspani L, Raimondi F, Bordone G, Iapichino G, Mancebo J, Guérin C, Ayzac L, Blanch L, Fumagalli R, Tognoni G, Gattinoni L, Prone-Supine II Study Group (2009). Prone positioning in patients with moderate and severe acute respiratory distress syndrome: a randomized controlled trial. JAMA..

[8] Munshi L, Del Sorbo L, Adhikari NKJ, Hodgson CL, Wunsch H, Meade MO (2017). Prone position for acute respiratory distress syndrome. A systematic review and meta-analysis. Ann Am Thorac Soc.

[9] Gattinoni L, Taccone P, Carlesso E, Marini JJ (2013). Prone position in acute respiratory distress syndrome. Rationale, indications, and limits. Am J Respir Crit Care Med.

[10] Gattinoni L, Tognoni G, Pesenti A, Taccone P, Mascheroni D, Labarta V, Malacrida R, di Giulio P, Fumagalli R, Pelosi P, Brazzi L, Latini R (2001). Effect of prone positioning on the survival of patients with acute respiratory failure. N Engl J Med.

[11] Villar J, Sulemanji D, Kacmarek RM (2014). The acute respiratory distress syndrome: incidence and mortality, has it changed?. Curr Opin Crit Care.

[12] Menegueti MG, Ardison KMM, Bellissimo-Rodrigues F, Gaspar GG, Martins-Filho OA, Puga ML, Laus AM, Basile-Filho A, Auxiliadora-Martins M (2015). The impact of implementation of bundle to reduce catheter-related bloodstream infection rates. J Clin Med Res.

[13] Miller DL, O’Grady NP, Society of Interventional Radiology (2012). Guidelines for the prevention of intravascular catheter-related infections: recommendations relevant to interventional radiology for venous catheter placement and maintenance. J Vasc Interv Radiol.

[14] Zingg W, Imhof A, Maggiorini M, Stocker R, Keller E, Ruef C (2009). Impact of a prevention strategy targeting hand hygiene and catheter care on the incidence of catheter-related bloodstream infections. Crit Care Med.

[15] O’Grady NP, Alexander M, Burns LA, Dellinger EP, Garland J, Heard SO (2011). Guidelines for the prevention of intravascular catheter-related infections. Am J Infect Control.

[16] Ranieri VM, Rubenfeld GD, Thompson BT, Ferguson ND, Caldwell E, ARDS Definition Task Force (2012). Acute respiratory distress syndrome: the Berlin Definition. JAMA..

[17] Timsit J-F, Rupp M, Bouza E, Chopra V, Kärpänen T, Laupland K, Lisboa T, Mermel L, Mimoz O, Parienti JJ, Poulakou G, Souweine B, Zingg W (2018). A state of the art review on optimal practices to prevent, recognize, and manage complications associated with intravascular devices in the critically ill. Intensive Care Med.

[18] Rijnders BJA, Van Wijngaerden E, Peetermans WE (2002). Catheter-tip colonization as a surrogate end point in clinical studies on catheter-related bloodstream infection: how strong is the evidence?. Clin Infect Dis.

[19] Toptas M, Sengul Samanci N, Akkoc İ, Yucetas E, Cebeci E, Sen O (2018). Factors affecting the length of stay in the intensive care unit: our clinical experience. Biomed Res Int.

[20] Hu SL, He HL, Pan C, Liu AR, Liu SQ, Liu L (2014). The effect of prone positioning on mortality in patients with acute respiratory distress syndrome: a meta-analysis of randomized controlled trials. Crit Care.

[21] Papazian L, Forel J-M, Gacouin A, Penot-Ragon C, Perrin G, Loundou A, Jaber S, Arnal JM, Perez D, Seghboyan JM, Constantin JM, Courant P, Lefrant JY, Guérin C, Prat G, Morange S, Roch A (2010). Neuromuscular blockers in early acute respiratory distress syndrome. N Engl J Med.

[22] Parienti J-J, Mongardon N, Mégarbane B, Mira J-P, Kalfon P, Gros A, Marqué S, Thuong M, Pottier V, Ramakers M, Savary B, Seguin A, Valette X, Terzi N, Sauneuf B, Cattoir V, Mermel LA, du Cheyron D (2015). Intravascular complications of central venous catheterization by insertion site. N Engl J Med.

[23] Kaur M, Gupta V, Gombar S, Chander J, Sahoo T (2015). Incidence, risk factors, microbiology of venous catheter associated bloodstream infections--a prospective study from a tertiary care hospital. Indian J Med Microbiol.

[24] Bell T, O’Grady NP (2017). Prevention of central line–associated bloodstream infections. Infect Dis Clin N Am.

[25] Timsit J-F, Schwebel C, Bouadma L, Geffroy A, Garrouste-Orgeas M, Pease S, Herault MC, Haouache H, Calvino-Gunther S, Gestin B, Armand-Lefevre L, Leflon V, Chaplain C, Benali A, Francais A, Adrie C, Zahar JR, Thuong M, Arrault X, Croize J, Lucet JC, Dressing Study Group (2009). Chlorhexidine-impregnated sponges and less frequent dressing changes for prevention of catheter-related infections in critically ill adults: a randomized controlled trial. JAMA..

[26] Mimoz O, Lucet J-C, Kerforne T, Pascal J, Souweine B, Goudet V, Mercat A, Bouadma L, Lasocki S, Alfandari S, Friggeri A, Wallet F, Allou N, Ruckly S, Balayn D, Lepape A, Timsit JF (2015). Skin antisepsis with chlorhexidine-alcohol versus povidone iodine-alcohol, with and without skin scrubbing, for prevention of intravascular-catheter-related infection (CLEAN): an open-label, multicentre, randomised, controlled, two-by-two factorial trial. Lancet..

[27] Carsetti A, Damia Paciarini A, Marini B, Pantanetti S, Adrario E, Donati A (2020). Prolonged prone position ventilation for SARS-CoV-2 patients is feasible and effective. Crit Care.

